# Effects of freeze–thaw on bank soil mechanical properties and bank stability

**DOI:** 10.1038/s41598-024-60698-z

**Published:** 2024-04-29

**Authors:** Zhen Yang, Xianyou Mou, Honglan Ji, Zhihao Liang, Jianghao Zhang

**Affiliations:** College of Water Resources and Civil Engineering, Inner Mongolia Agricultural University, Hohhot, 010018 Inner Mongolia China

**Keywords:** Freeze–thaw cycles, Freezing temperature, Soil mechanical properties, Bank stability, Inner Mongolia section of the Yellow River, Mechanical properties, Hydrology, Riparian ecology

## Abstract

Riverbank instability in the seasonally frozen zone is primarily caused by freeze–thaw erosion. Using the triaxial freeze–thaw test on the bank of Shisifenzi Bend in the Yellow River section of Inner Mongolia, we investigated the changes in the mechanical properties of the soil at different freezing temperatures and freeze–thaw times, and analyzed the bank’s stability before and after freezing based on the finite element strength reduction method. The results showed that the elastic modulus, cohesion, internal friction angle and shear strength of the soil tended to decrease with the increase in the number of freeze–thaw cycles and the decrease in freezing temperature. After 10 freezing cycles at − 5 ℃, − 10 ℃, − 15 ℃ and − 20 ℃, the modulus of elasticity of soil decreased by 40.84 ~ 68.70%, the cohesion decreased by 41.96 ~ 56.66%, the shear strength decreased by 41.92 ~ 57.32%, respectively. Moreover, the stability safety coefficient of bank slope decreased by 18.58% after freeze–thaw, indicating that the freeze–thaw effect will significantly reduce the stability of bank slope, and the bank slope is more likely to be destabilized and damaged after freeze–thaw.

## Introduction

The Inner Mongolia section of the Yellow River is at the northernmost part of the Yellow River. The winters here are cold and long, which is typical of a seasonal frozen soil region. The bank slope of the Inner Mongolia section of the Yellow River is affected by both water erosion and freeze–thaw erosion in winter. The freeze–thaw cycle refers to a physical phenomenon in which water in the soil undergoes phase changes between ice and water due to alternating positive and negative temperatures, which can severely damage the soil properties of the bank slope^1^ and increase the risk of a bank collapse, threatening the safety of coastal people and their property. Therefore, it is necessary to study the changes in soil properties and stability of bank under freeze–thaw.

The stability of riverbanks is influenced by various factors^2^, including hydraulic erosion, gravitational erosion, as well as the composition and properties of bank soil^3^. Although many studies have examined the stability of riverbanks under hydraulic action^4,5^, few have examined the effect of freeze–thaw to riverbank stability. It has been shown that freeze–thaw can damage soil structure and alter its physical and mechanical properties^6^. The physical properties of soil changes mainly in terms of soil density and permeability^7^. As a result of freezing and thawing, dense soil becomes loose and loose soil becomes dense, making them more permeable^8^. Additionally, the freeze–thaw action alters the mechanical parameters of soil elastic modulus^9^, cohesion, and internal friction angle^10,11^. Tang et al.^12^ found that the elastic modulus of expansive soil significantly decreased by up to 60% after one freeze–thaw cycle. Similarly, Lee et al.^13^ found that the elastic modulus of clay decreased by over 50% after experiencing freeze–thaw cycles. Meanwhile, Simonsen et al.^14^ research revealed that the change in initial elastic modulus of soil under freeze–thaw action is related to the particle size of the soil. It can be observed that there is a consensus among researchers regarding the decrease in soil elastic modulus after freeze–thaw cycles, but there is still no unified understanding of the pattern of freeze–thaw effects on cohesion and internal friction angle. Zhang et al.^15^ found a significant decrease in the shear strength of saline soil under freeze–thaw action. Wang et al.^16^ conducted experiments on clay and discovered that its strength initially increased and then decreased with the increase in freeze–thaw cycles, stabilizing after exceeding 10 cycles. However, Wang et al.^17^ argued that the decrease in cohesion of loess after experiencing freeze–thaw cycles is directly proportional to the number of cycles, and its internal friction angle increases with the increase in freeze–thaw cycles. Zhou et al.^1^ found that the strength, elastic modulus, and cohesion of loess all showed a decreasing trend with the increase in freeze–thaw cycles, reaching a critical value where these three mechanical parameters stabilized after surpassing this critical value. It can be observed that under freeze–thaw action, soil mechanical parameters exhibit varying changes, and similarly, the patterns of mechanical characteristic changes in soil differ under different freezing temperatures. Some researchers^18^ have found that the lower the freezing temperature, the poorer the mechanical properties of freeze-thawed soils. Additionally, the lower the freezing temperature, the lower the cohesion of the soil after freezing and thawing, and the greater the proportion of reduction, while the internal friction angle does not change significantly. Wang et al.^19^ found through triaxial test the static strength and initial tangential modulus of an improved cohesive soil with a high liquid limit decreased with the decrease in freezing temperature under the condition of triple freezing from − 2 to − 20 °C. As a result of the triaxial test of saturated primary pulverized clay, Yu et al.^20^ found that the soil cohesion decreased and internal friction angle increased, and the soil cohesion and internal friction angle gradually decreased with a decrease in the freezing temperature when the freezing temperature was higher than − 15 °C. Chang et al.^21^ found that the triaxial test of powdered sandy soil did not significantly change the mechanical properties of soil at different freezing temperatures. Overall, these studies indicate that the strength of soil after freeze–thaw cycles exhibits both strengthening and weakening effects, and the deterioration rate varies with decreasing freezing temperature, showing patterns of increase, decrease, or no effect.

Research on riverbank stability is currently primarily based on the models proposed by Osman and Thorne^22^, Osman and Thorne initially developed bank stability models in the early 1980s based on the method of limit equilibrium, considering the effects of river erosion on bank boundaries^22^. In the 1990s, Dandy et al.^23^ improved the Osman and Thorne bank stability analysis model by considering the effects of static water pressure and pore water pressure. Subsequently, domestic and foreign scholars have considered factors such as multi-layered bank slopes^24–26^, changes in flood levels^27^, groundwater levels^28^, and pore water pressure^29^ on bank stability. However, research on the effects of freeze–thaw action on bank stability is limited. Preliminary studies by Jia et al.^30^ considered freeze–thaw action and established a model for bank collapse instability, finding that freeze–thaw action can affect bank slope stability, but only considered the parallel retreat process of the beach bank. Qin et al.^31^ further discovered that freeze–thaw cycles significantly affect the instability of soil bank slopes, with a decrease in safety factor of 10.43% after 5 freeze–thaw cycles, directly leading to slope failure. However, there are differences in the freezing temperatures experienced by soil at different depths. Currently, there is a lack of research on the stability changes of bank slopes under different freezing temperatures. Therefore, it is necessary to further study the changes in beach bank stability and instability mechanisms under freeze–thaw action.

In summary, freeze–thaw will significantly alter soil properties, thereby affecting bank stability. However, the law of change in soil properties at different freezing temperatures is not uniform, and the freezing temperatures of soil at various depths differ. Thus, it is necessary to examine the law of change in soil properties of the bank at different freezing temperatures and their influence on bank stability. Therefore, this study investigates the changes in soil mechanical parameters resulting from different freezing temperatures as well as the number of freezing and thawing times by transforming the triaxial soil samples of the bank and performing triaxial tests several times at different freezing temperatures following freezing and thawing. In addition, the stability of the bank before and after freezing and thawing is simulated using the strength reduction method and the finite element software ABAQUS to provide a theoretical basis and technical guidance for the construction and maintenance of the bank in the seasonally frozen zone.

## Materials and methods

### Study area overview

**Figure 1:**
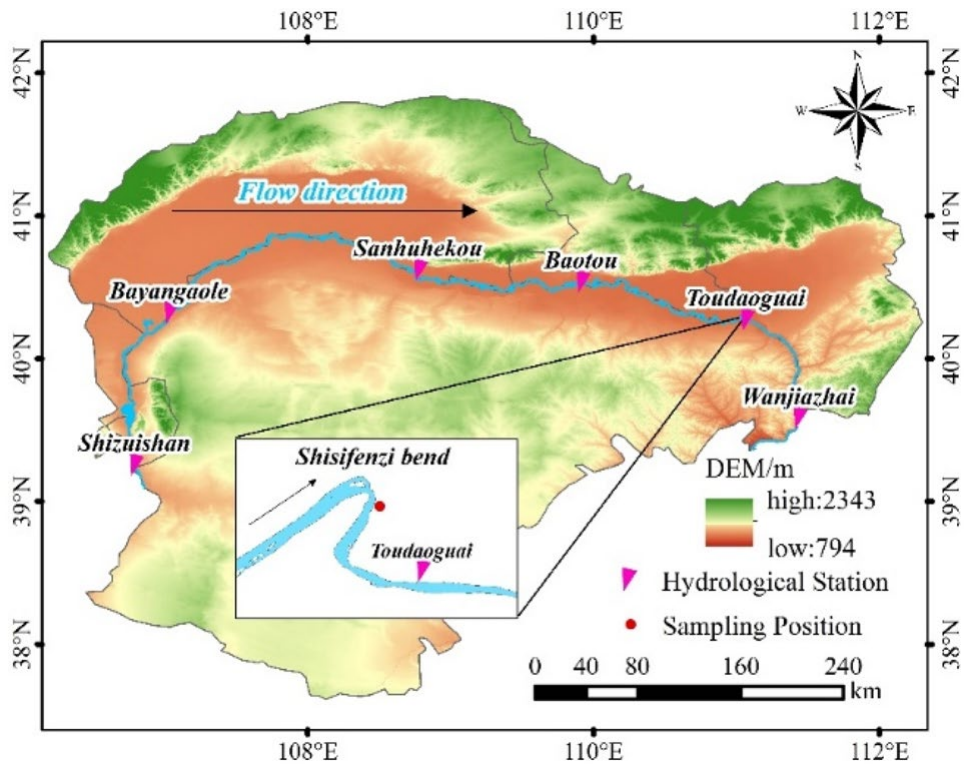
Location map of the study area. This figure is generated by QGIS3.32.3(http://qgis.org/en/site/).

The study area selects the Shisifenzi parts of the Yellow River in Inner Mongolia. Using QGIS3.32.3 software, firstly, the vector data of the Inner Mongolia section of the Yellow River Basin is obtained by intersecting the area vector data of the Yellow River Basin and the Inner Mongolia Autonomous Region downloaded from the National Geographic Basic Information Center (https://www.ngcc.cn/ngcc/html/1/396/397/16118.html). Then, the vector data is used to crop the DEM data downloaded from the geospatial data cloud (http://www.gscloud.cn/) to obtain the geographic elevation data of the study area, and then the geographic location information of the hydrological station is inserted to obtain the location map of the study area. As shown in Fig. 1. which is located in Tuoketuo County at 40°17ʹ39 ʺN, 111°2ʹ53 ʺE, and the altitude is about 997 m. The river flow sharply direction from northeast to southwest, with a bending angle of nearly 160°, a bending coefficient of 3.23, and a specific gradient of about 0.1‰, with a width of 200–500 m. The northwest winds prevail in the study area all year round, and the average wind speed of about 3 ~ 4 m/s. There is an average annual rainfall of 359.8 mm and an average annual evaporation of 1849 mm in the area. Moreover, the annual maximum temperature is 38.4 °C, the minimum temperature is − 36.3 °C, the annual average temperature is 7.3 °C, the temperature drops below 0 °C around mid-November, and the temperature rises above 0 °C at the end of March of the following year. Furthermore, the freeze–thaw period lasts approximately four months, for about 130 days, which is typical for a seasonally frozen area.

### Soil sample and test schemes

#### Soil sample

**Figure 2:**
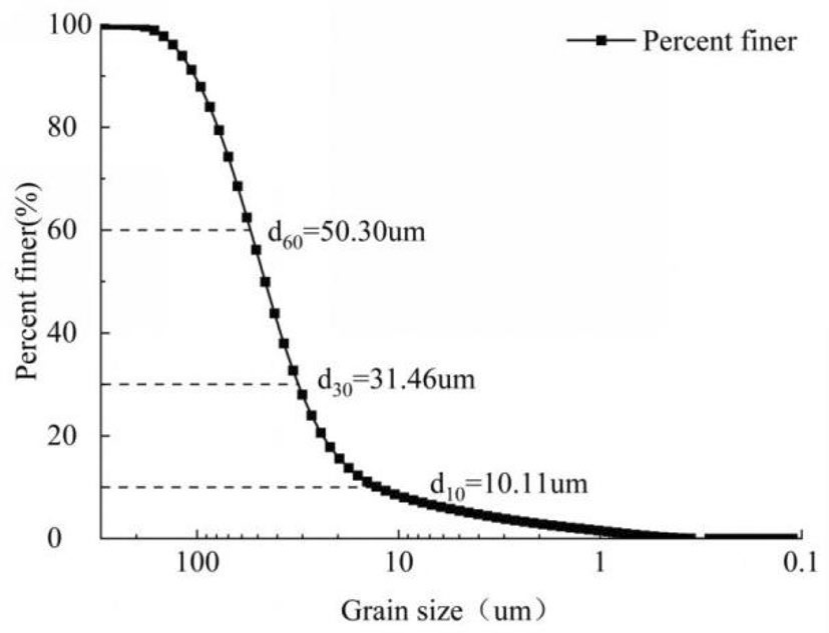
The grain grading curve of the soil sample.

As the study area freeze–thaw zone of about 1.6 m depth in winter, and there are grass and gravel on the surface of the bank slope. Therefore, the surface debris should be removed and soil samples should be collected from a depth of 0.1 m ~ 1.6 m. Table 1 shows the basic physical properties of the soil measured according to the geotechnical test method Standard^32^. Figure 2 presents the grain gradation curve of the soil. Based on the inhomogeneity coefficient C_u_ = 5.37 and the curvature coefficient C_c_ = 1.8, the soil sample is well graded, and according to the Engineering Classification Standard for Soil, it is classified as a silt soil with low liquid limit.

**Table 1:** Basic physical properties of our soil sample.

Maximum dry density	Optimum moisture content	Specific gravity	Liquid limit	Plastic limit	Plasticity index $I_{P}$	Liquidity index $I_{L}$
1.58g/cm^3^	18.48%	2.7	29.14%	23.69%	5.45	0.24

#### Sample preparation

**Figure 3:**
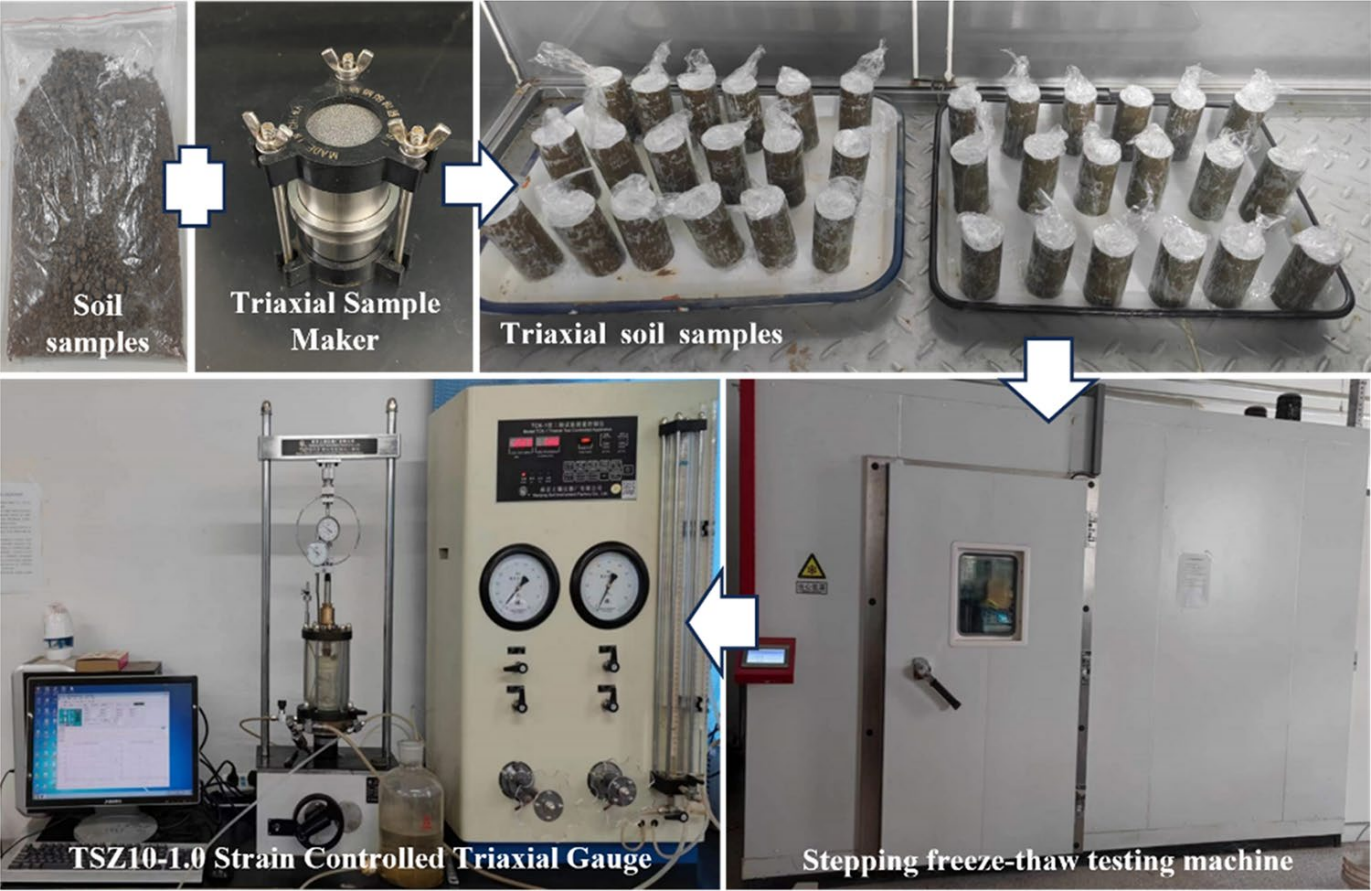
Test procedure and instrumentation.

As it was difficult to collect and store the intact soil samples, remolded soil samples had to be prepared for testing in accordance with the geotechnical test method standard^32^, The test procedure and apparatus are shown in Fig. 3.From the study area, bank soil was collected from a depth of 0.1–1.6 m, and it was then dried, crushed, and passed through a sieve of 2 mm. This test mainly explores the change of soil characteristics under different freezing temperatures, so the test does not involve the change of water content of soil samples. Therefore, the soil samples with an optimal water content was prepared for the test, and the prepared soil sample was sealed for 24 h. Once the water had fully equilibrated, the samples were compacted into five layers using a triaxial sample maker according to the geotechnical test specifications to produce a cylindrical triaxial sample (height 80 mm, diameter 39.1 mm) was made. Seal with cling film to prevent moisture loss.

The prepared soil samples were then put into the walk-in freeze–thaw chamber (Fig. 3). This study focuses on the changes in soil properties at different freezing temperatures. Therefore, the triaxial samples were frozen at − 5 °C, − 10 °C, − 15 °C, and − 20 °C for 12 h, then thawed at 15 °C for 12 h as one freeze–thaw cycle. According to previous research^16^, soil properties stabilize after approximately 10 freeze–thaw cycles, so we chose 1, 3, 5, 7, and 10 freeze–thaw cycles, with 0 freeze–thaw samples as the control group.

#### Triaxial shear test

**Figure 4:**
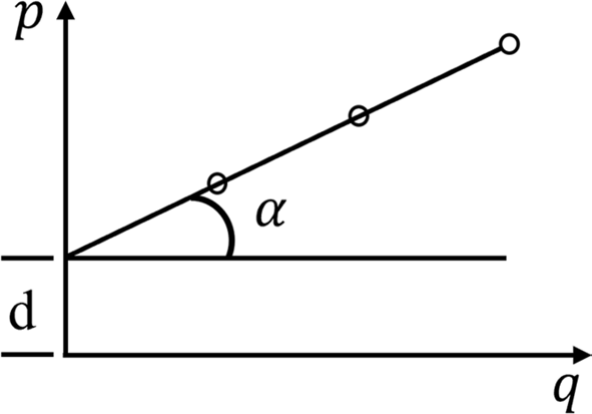
Diagram of principal stress line of soil failure.

This study utilised a TSZ10-1.0 stress-controlled triaxial gaugu (Fig. 3). The soil in the study area comprises perennially deposited silt, which consolidates under gravity during the formation process. Since the bank has a short damage time, and it can be assumed that the water in the bank has not been transported, we selected the consolidation-undrained test (CU), with the ambient pressure as 50 kPa, 100 kPa, and 150 kPa, and a shear rate of 0.08 mm/s. The shear test continued until the peak force gauge reading was reached or until the axial strain reached 15%. If the peak force gauge reading did not appear, the axial strain was set to 15% of the partial stress value for failure strength.^32^. With $p = \left( {\sigma 1 - \sigma 3} \right)/2$ as the vertical coordinate and $q = \left( {\sigma 1 + \sigma 3} \right)/2$ as the horizontal coordinate, Fig. 4 illustrates how the main line of damage is plotted for the soil’s at different circumferential pressures, where the angle of inclination of the main line is α and the point of intersection with the vertical axis is d. Then, the friction angle and cohesion within the soil can be calculated by formula (1) and formula (2), and then the shear strength is calculated by formula (3–5)^32^.

1 $$\varphi = \sin^{ - 1} \left( {\tan \alpha } \right)$$

2 $$c = \frac{d}{\cos \varphi }$$

3 $$\tau = c + \sigma \tan \varphi$$

4 $$\sigma = \frac{{\sigma_{1} + \sigma_{3} }}{2} + \frac{{\sigma_{1} - \sigma_{3} }}{2}\cos 2\alpha$$

5 $$\alpha = 45^\circ + \frac{\varphi }{2}$$

where: φ is the bank soil internal friction angle, °. c is the bank soil cohesion, kPa. τ is the shear strength of soil, kPa. σ is the normal stress at the damaged surface of the soil, kPa. α is the angle of the damaged surface, °.

The modulus of elasticity is an important parameter for analyzing the stability of bank slopes. The soil stress–strain curve mainly consists of elastic deformation phase and plastic deformation phase. At the beginning of loading, the soil sample undergoes elastic deformation, and the stress increases linearly with strain, at which point the slope of the curve represents the initial elastic modulus of the soil. Based on the triaxial test results and previous studies^33^, we have considered the ratio of the corresponding stress increment to the axial strain increment at 1.0% of the axial strain as the elastic modulus E of the soil:

6 $$E = \frac{{\sigma_{1.0\% } - \sigma_{0} }}{{\varepsilon_{1.0\% } - \varepsilon_{0} }}$$

where: $\varepsilon_{0}$ and $\varepsilon_{1.0\% }$ are the initial axial strain and the axial strain at 1.0%, respectively.$\sigma_{0}$ and $\sigma_{1.0\% }$ are the initial axial stress and the axial stress with axial strain of 1.0%, respectively.

## Results

### Soil stress–strain relationship at different freezing temperatures

**Figure 5:**
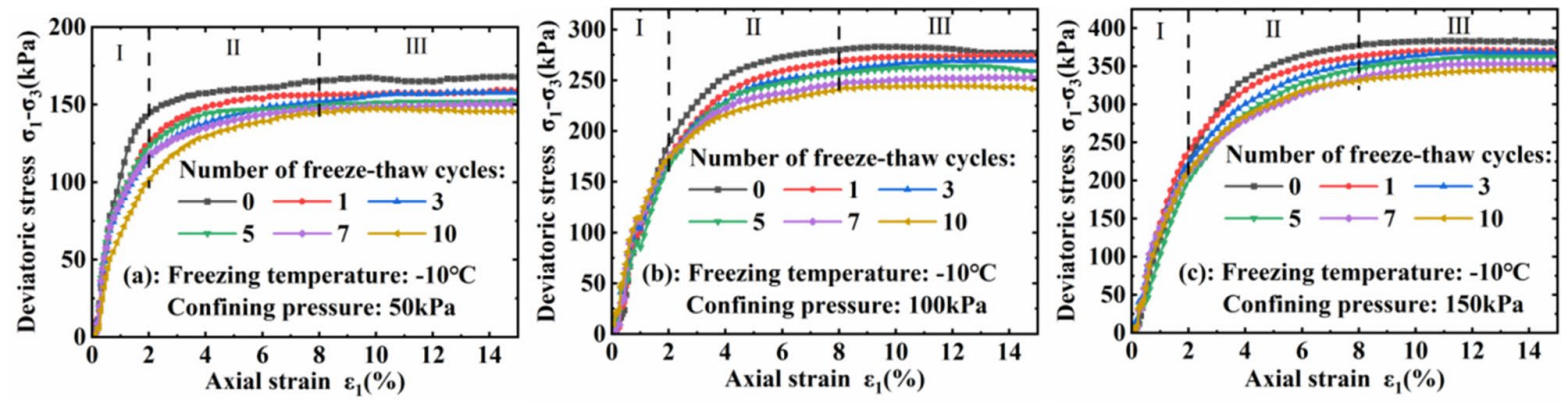
Soil stress–strain relationship curves at different freezing temperatures.

The stress–strain curves of soils under different freezing temperatures and confining pressures exhibot the same pattern of changes, so we analyze the stress–strain curve of frozen soil at − 10 °C as an example (Fig. 5). At high envelope pressures, soil stress–strain relationships can be categorised into two types: “strain stabilized” and “weakly consolidated”. These curves typically exhibit three major deformation stages: (I) Linear-elastic deformation stage, which occurs between 0 and 2% of axial strain, and where the soil stress increases linearly with strain, primarily due to the high cementation strength between the soil particles and the strong deformation resistance at this stage. (II) Plastic consolidation deformation stage, which occurs between 2 and 8% of axial strain, and when the soil structure is damaged, the Stress tends to increase with increasing strain, but the slope of the curve gradually decreases, and plastic deformation occurs. (III) Stress stabilization stage, where the axial strain is between 8 and 15% when the soil structure has been damaged, the soil particles are no longer compressed with the increase in axial stress, and the stress does not vary with the change in strain and the slope of the curve tends to zero.

### Effects of freeze–thaw action on soil mechanical parameters

#### Elastic modulus

**Figure 6:**
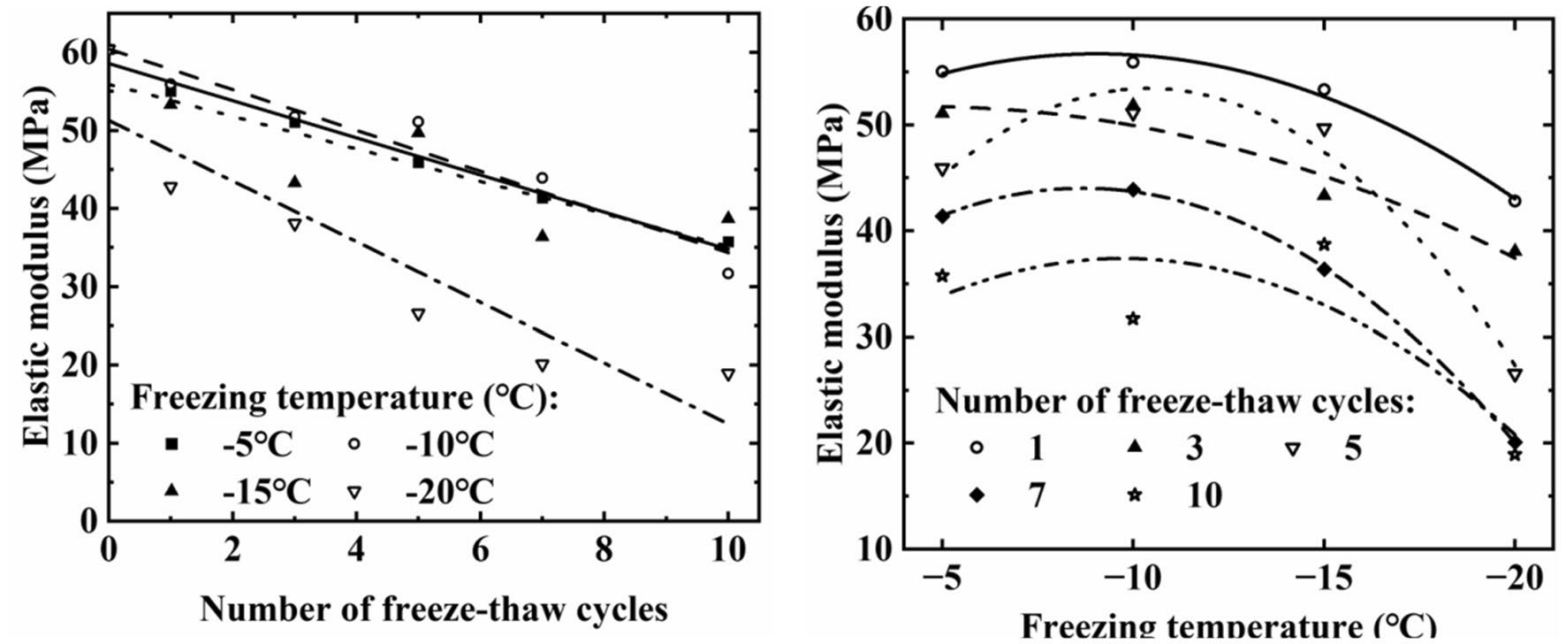
Variation of soil elastic modulus under different freezing temperatures and freeze–thaw times.

Figure 6 illustrates the changes in soil elastic modulus under different freeze–thaw cycle numbers and freezing temperatures. Overall, the soil’s elastic modulus showed a decreasing trend with increasing number of freeze–thaw cycles, decreasing by 40.84%, 47.53%, 35.96%, and 68.70% after 10 freeze–thaw cycles at − 5 °C, − 10 °C, − 15 °C, and − 20 °C, respectively. In addition, as the freezing temperature decreases, the elastic modulus of the soil first increases slightly and then decreases. This is because during the freeze–thaw cycle, the volume change caused by the freeze–thaw of the pore water inside the soil will change the arrangement and connection of the soil particles, resulting in the degradation of the cementation between the soil particles, and the connection between the soil particles. The cementation is the main source of the elastic modulus of the soil, so the elastic modulus of the soil will be greatly reduced after the freeze–thaw cycle.

#### Cohesion and internal friction angle

##### Effect of the number of freeze–thaw cycles on soil cohesion and internal friction angle

**Figure 7:**
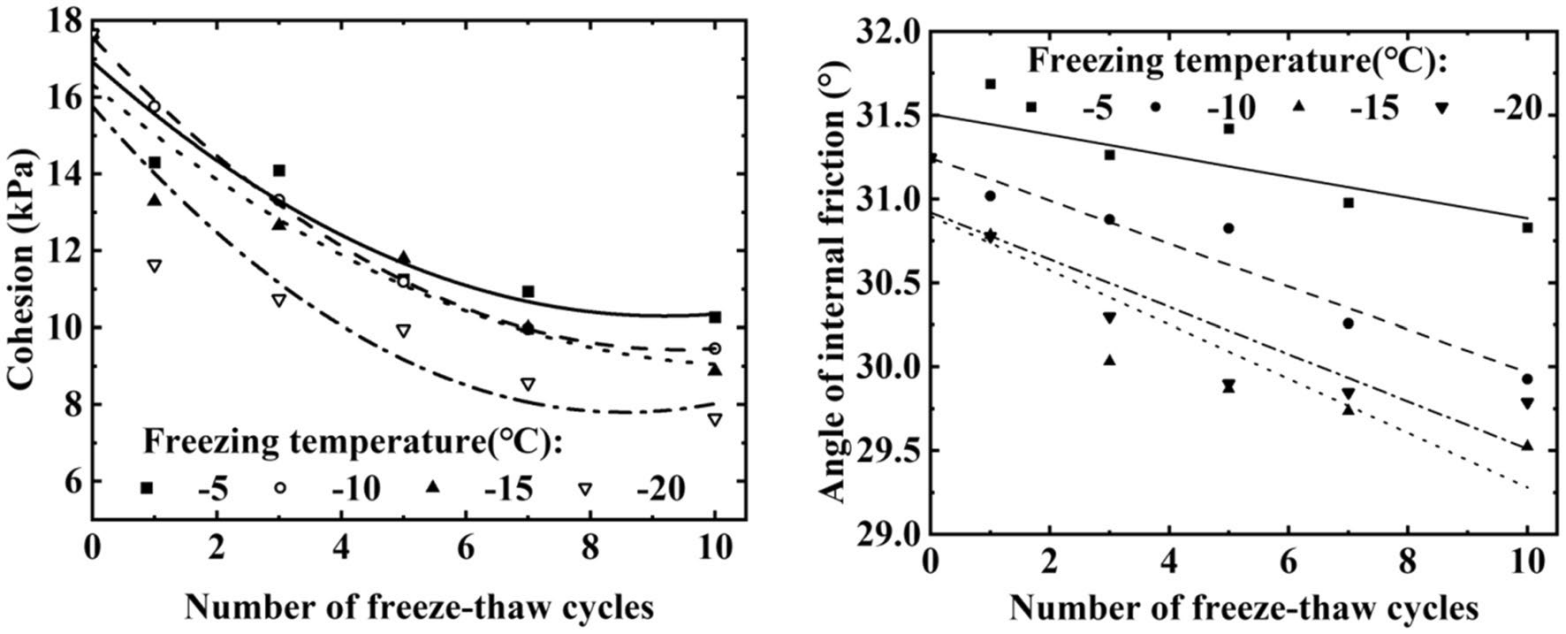
Variation of cohesion and internal friction angle of soil under different freeze–thaw times.

Figure 7 depicts the impact of varying freeze–thaw cycles numbers on soil cohesion and angle of internal friction. Both soil cohesion and internal friction angle tendency to decrease with an increasing number of freeze–thaw cycles. After 10 freezes-thaws cycles, soil cohesion decreased by 41.96%, 45.94%, 49.69%, and 56.66% after freezing at temperatures of − 5 °C, − 10 °C, − 15 °C, and − 20 °C, respectively. The soil has a porous structure based on the loose skeleton of the particles. Freezing and thawing processes damages the soil structure, resulting in larger soil pores and the breakdown of cementation between the soil particles, reducing the frictional force. Since soil cohesion mainly results from the friction and cementation between the soil particles, freezing and thawing significantly reduces it.

##### Effect of the freezing temperatures on soil cohesion and internal friction angle

**Figure 8:**
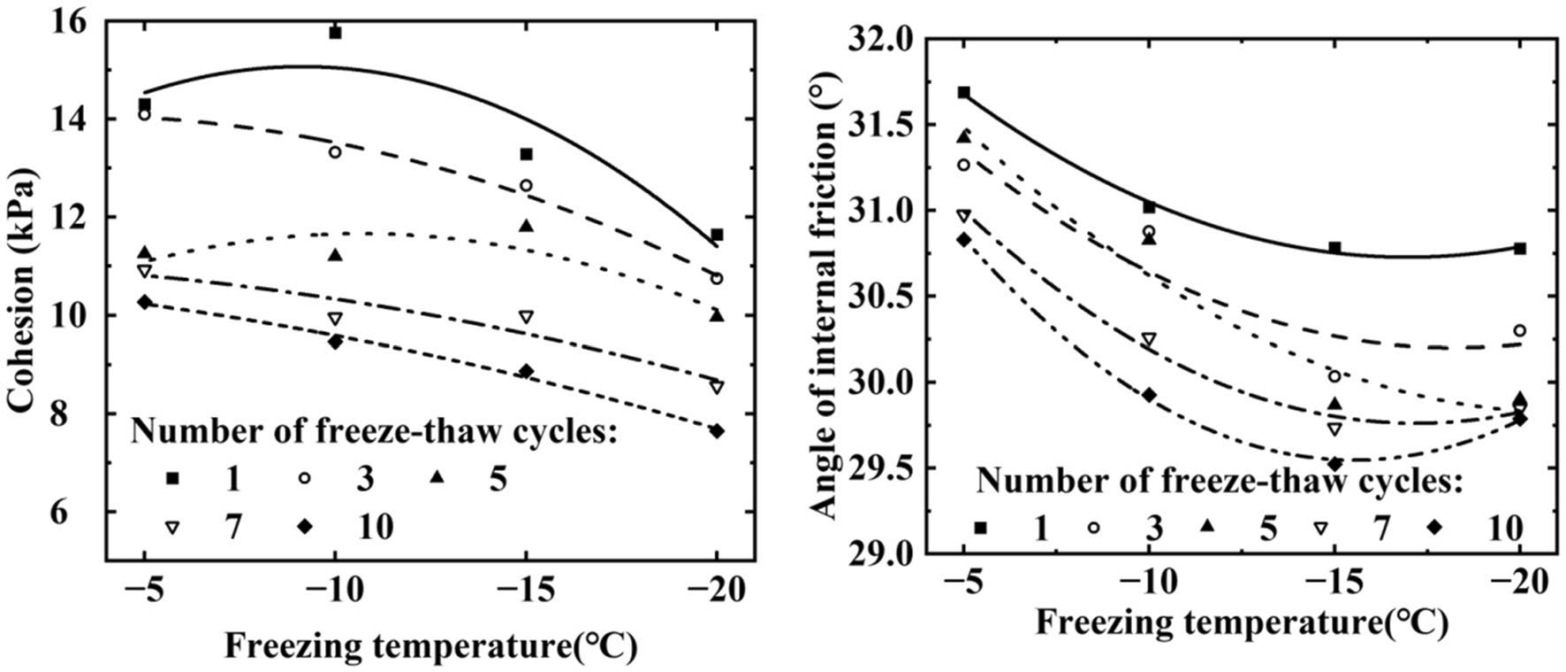
Variation of cohesion and internal friction angle of soil under different freeze–thaw temperatures.

Figure 8 illustrates the effect of different freezing temperatures on soil cohesion and internal friction angle. It is evident that as the freezing temperature decreases, the cohesion of the soil also decreases, indicating that the freeze–thaw cycle weakens soil cohesion, with the effect becoming more pronounced at lower freezing temperatures. As the freezing temperature decreases, the internal friction angle of the soil gradually decreases and finally stabilizes. Analysis shows that the soil consists of solid matter, liquid and gas three components, when the soil freezes, part of the soil moisture will freeze to form ice crystals, the existence of ice crystals will produce freezing and expansion damage to the soil structure, so that the pore space between the soil particles becomes larger, which destroys the bonding ability between the soil particles, resulting in a decrease in the cohesion and the angle of internal friction. And the lower the freezing temperature, the more water molecules inside the soil body are converted into ice crystals, and the ice crystals damage the soil structure more seriously. Therefore, the weakening effect on soil cohesion is more significant at lower freezing temperatures.

#### Shear strength

**Figure 9:**
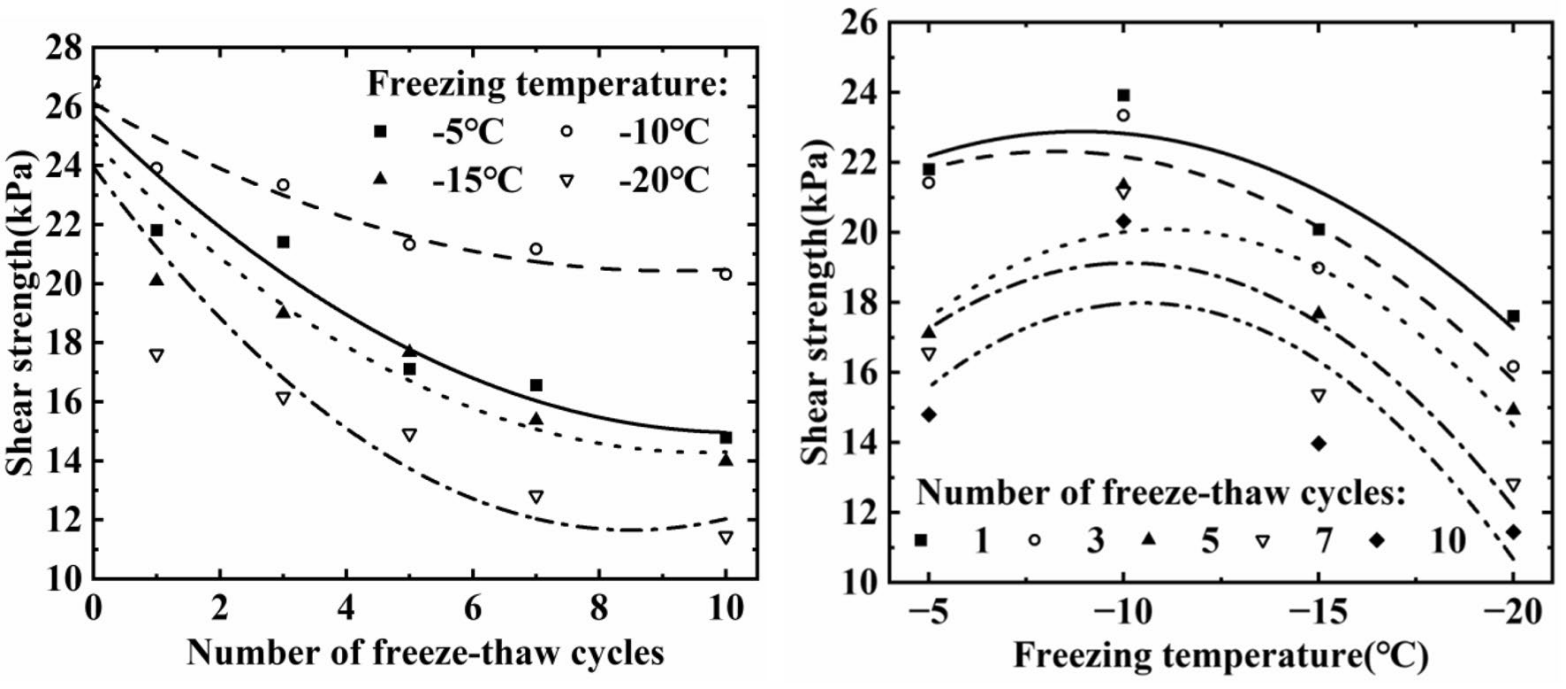
Variation of soil shear strength under different freeze–thaw times and freeze–thaw temperatures.

Figure 9 illustrates the soil shear strength as affected by freeze–thaw cycles and the freezing temperature. As the number of freeze–thaw cycles increases, the shear strength of the soil gradually decreases and is basically stable after about seven freeze–thaw cycles. The shear strength of the soil decreased by 41.92%, 46.25%, 50.54% and 57.32% after 10 freeze–thaw cycles at − 5 °C, − 10 °C, − 15 °C and − 20 °C, respectively. It shows that the lower the freezing temperature, the greater the decrease in the shear strength of the soil, and when the freezing temperature is − 10 °C, the soil strength is the largest. Shear strength is the maximum capacity of soil to resist shear damage. Shear strength is calculated by soil cohesion and the angle of internal friction, and we already know that the soil cohesion and the angle of internal friction decrease after freeze-thawing, so the soil shear strength gradually decreases under the action of freeze-thawing cycle, and the lower the freezing temperature, the greater the decrease of soil strength.

### Simulation analysis of bank stability under different freezing temperatures

#### Calculation method

In geotechnical engineering analysis, due to the nonlinearity of geotechnical constitutive relations and the complexity of load and boundary conditions, numerical methods are usually used for calculation. ABAQUS software contains rich material models, element models, loads and boundary conditions. It is good at solving nonlinear problems and is suitable for geotechnical engineering analysis^34^. Therefore, Using the finite element software ABAQUS, the stability of the bank under freeze–thaw action was determined by the shear strength reduction technique. The shear strength reduction technique divides the bank soil cohesion c and internal friction angle φ by the discount factor F according to Eqs. (7) and (8) to obtain a new set of cʹ and φʹ values as the new material parameters for the experimental calculation^7^. Additionally, the curve of the displacement versus the discount factor at the characteristic point of the slope starts to change abruptly, which is an unstable criterion. At this point, the *F*r is the minimum stability factor of the bank, and the bank has reached its final state of damage.

7 $$c^{\prime } = \frac{c}{{F_{r} }}$$

8 $$\varphi^{\prime } = {\text{arctan}}\frac{tan\phi }{{F_{r} }}$$

where: c is the cohesion that the soil can provide, kPa; $\phi$ is the internal friction angle that the soil can provide, °; cʹ is the cohesive force required to maintain equilibrium, kPa; φʹ is the angle of internal friction required to maintain equilibrium, °; *F*r is the strength discount factor.

#### Model construction

**Figure 10:**
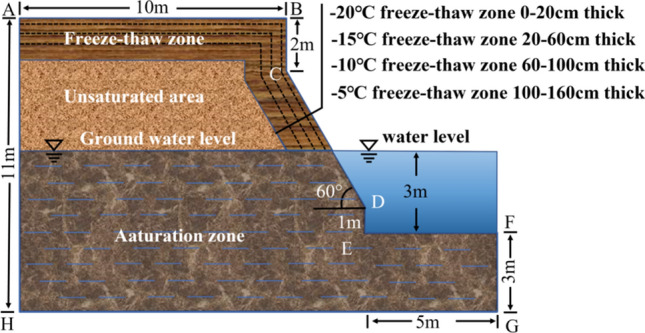
Bank slope profile diagram.

A study of the bank of the Yellow River in Inner Mongolia revealed that the natural bank is about 6 to 10 m high. The upper and lower parts of the bank are about 2 m and 1 m steep, respectively, and subject to water erosion, the slope of the bank is about 30° ~ 80°, the slope of the bank in the curve is about 60° ~ 80°. Hence, the slope inclination is generalized to a height of 8 m, the upper and lower parts of the slope are simplified to an upright slope with a height of 2 m and 1 m, respectively, and the slope inclination is set to 60°. The calculation of the slope is shown in Fig. 10. To facilitate the calculation, assumed that the soil on the bank slope is homogeneous, that the change in river level is not considered. Before freezing and thawing, the soil on the bank slopes is assumed to be saturated below the river level and unsaturated above the river level. After freezing and thawing, the soils below the freezing and thawing zone are assumed to be saturated due to the transport of soil moisture to the freezing front during freezing.

**Figure 11:**
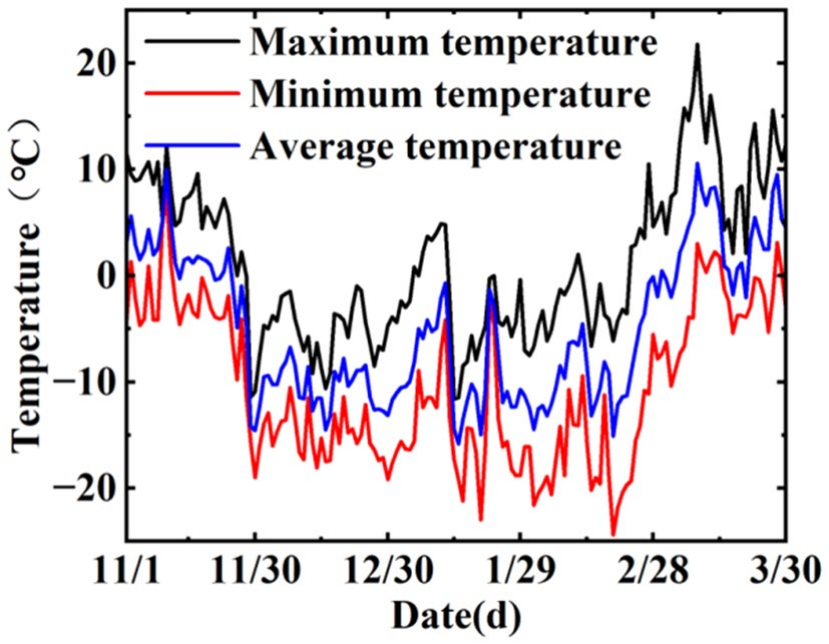
Winter temperature change in Shisifenzi from 2022 to 2023.

**Figure 12:**
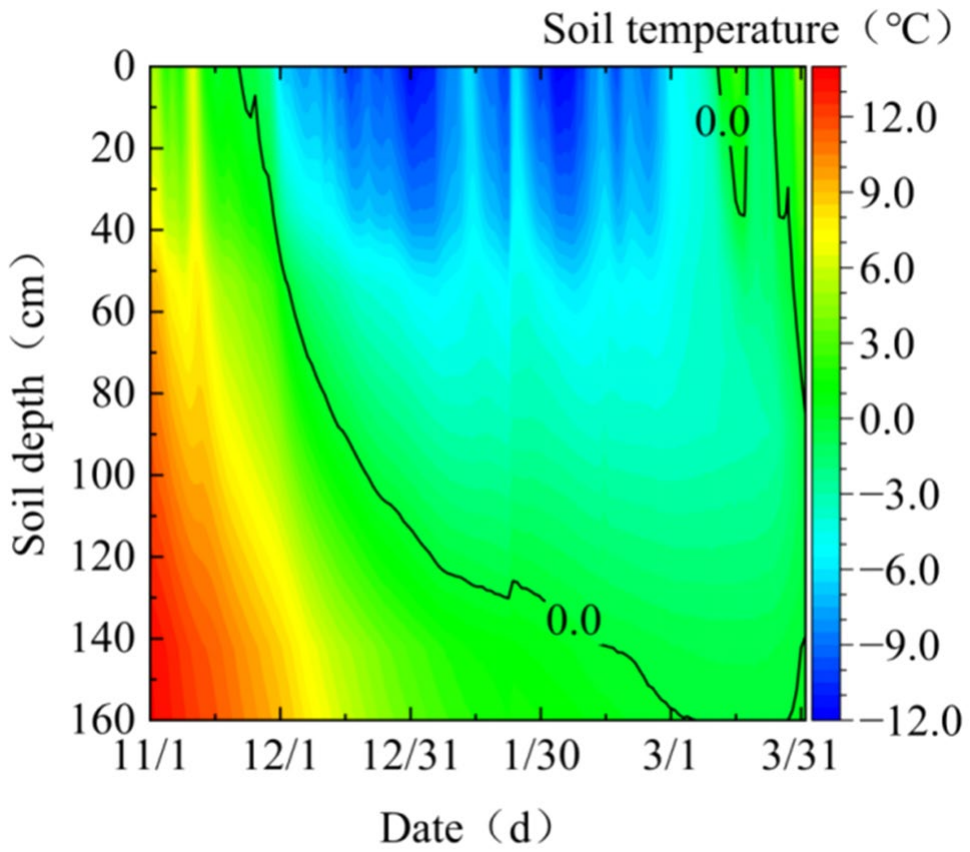
Temperature change of bank soil in Shisifenzi bend from 2022 to 2023.

By monitoring the temperature of the study area from November 2022 to March 2023 (Fig. 11) and the temperature of soil at different depths of the riparian slope (Fig. 12), it was found that the winter temperature in the study area changed drastically, with positive and negative daily temperature fluctuations, and the maximum daily temperature difference was nearly 20 °C. As the soil temperature changed over time, it was “first falling and then rising, slowly freezing and rapidly melting”. It was affected by its own thermal properties and exhibited some hysteresis and attenuation when undergoing temperature changes, so the freezing temperature of the soil at different depths also differed, and the cohesion and internal friction angle changes of soils at different freezing temperatures are found to be different through the previous study. Therefore, the area affected by frost is divided into four depth intervals of 0 cm ~ 20 cm, 20 cm ~ 60 cm, 60 cm ~ 100 cm, and 100 cm ~ 160 cm corresponding to − 5 °C,  − 10 °C,  − 15 °C, and − 20 °C, respectively. Soil sampling on the bank has shown that the natural soil capacity is approximately 18.1 kN/m^3^ and the saturation capacity is 19.3 kN/m^3^. In summary, the soil capacity of the embankment above and below the river level is 18.1 kN/m^3^, and 19.3 kN/m^3^, respectively. 0 cm ~ 20 cm, 20 cm ~ 60 cm, 60 cm ~ 100 cm and 100 cm–160 cm depth of the soil body corresponding to the unsaturated soil body at − 5 °C, − 10 °C, − 15 °C, and − 20 °C freezing temperature after 10 mechanical freeze–thaw parameters. The parameters used in the model calculations are shown in Table 2. Considering the difference between the remolded soil sample and the actual soil sample, the test parameters are reduced by calculating the parameters. The effective cohesion c is 0.7 times of the test value, and the effective internal friction angle is 0.9 times of the test value9^29^.

**Table 2:** Soil mechanics calculation parameters of bank model.

Soil sample	Specific weight $\gamma \left( {kN/m^{3} } \right)$	Elastic modulus $E\left( {MPa} \right)$	Poisson ratio $v$	Cohesion *c* ($kPa$)		Internal friction angle *ϕ* (°)	
				Test value* c*	Valid values* c*	Test value* ϕ*	Valid values* ϕ*
Saturated soil	1.81	60	0.25	17.65	12.355	31.25	28.125
Unsaturated soil	1.93	20	0.30	9.21	6.470	28.14	25.326
0–20cm deep F-T zone soil	1.81	40	0.27	7.65	5.355	29.79	26.811
20–60cm deep F-T zone soil	1.81	40	0.27	8.88	6.216	29.62	26.658
60–100cm deep F-T zone soil	1.81	40	0.27	9.54	6.678	30.74	27.666
100–160cm deep F-T zone soil	1.81	40	0.27	10.25	7.175	30.65	27.585

#### Analysis of bank stability under freeze–thaw action

##### Displacement change

**Figure 13:**
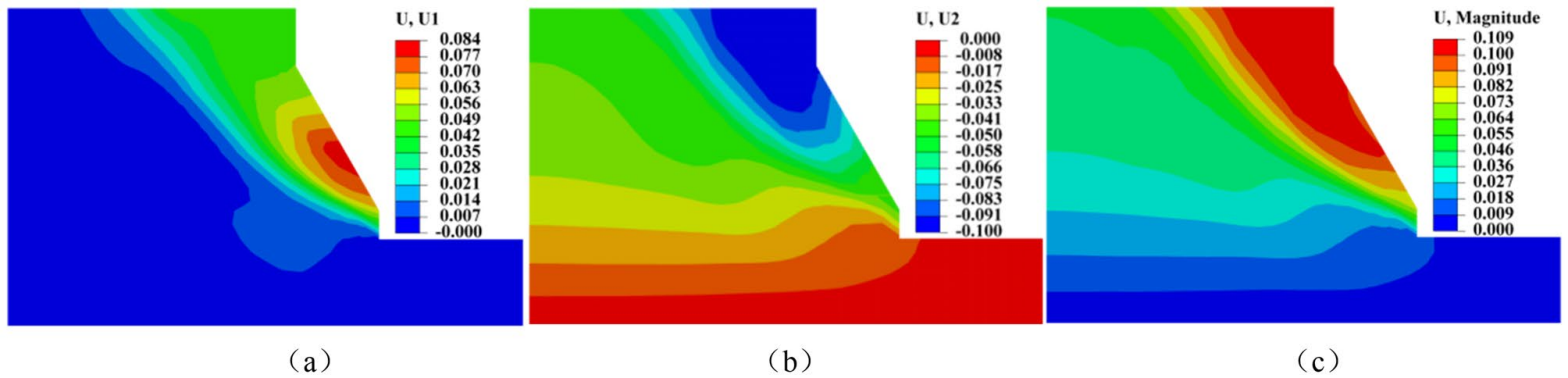
Displacement of bank before freeze and thaw. (**a**) Horizontal displacement (**b**) Vertical displacement (**c**) Maximum displacement.

**Figure 14:**
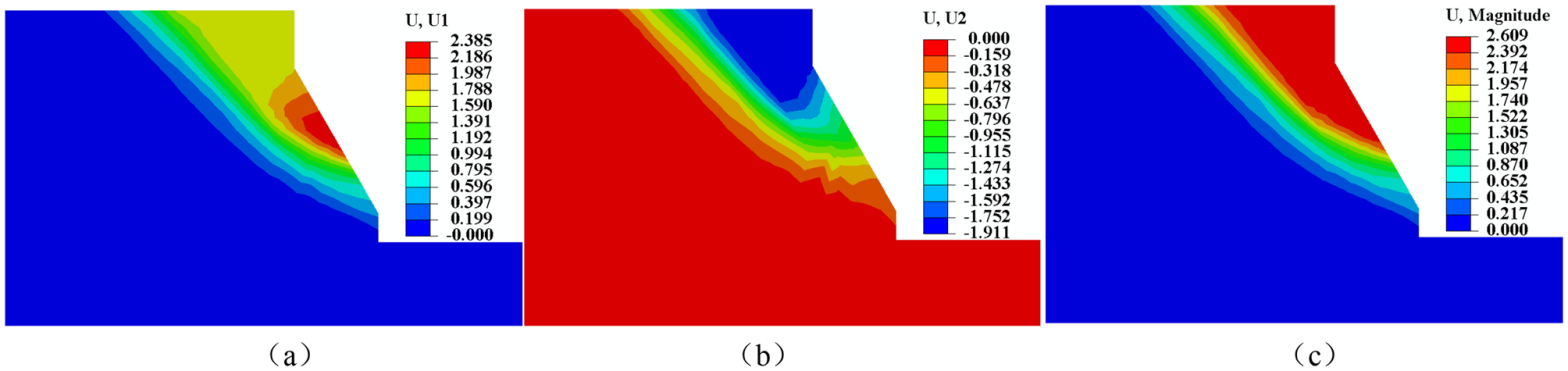
Displacement of bank after freeze and thaw. (**a**) Horizontal displacement (**b**) Vertical displacement (**c)** Maximum displacement.

Figures 13 and 14 illustrate the changes in bank slope displacement before and after freeze–thaw. The slope generally slides along a circular surface. Before freeze–thaw cycles, horizontal displacement of the bank slope is predominantly concentrated at the slope’s base, with the largest displacement occurring there. Soil settlement is mainly concentrated at the top of the slope, which makes the slope to slide along from the foot of the slope to the top and overall damage occurred. After freeze–thaw, the horizontal displacement of the bank slopes is mainly concentrated at the junction of the freeze-thawed and unfreeze-thawed zones, while the subsidence mainly occurs in the upper part of the slope, causing the slope to slide mainly along the freeze–thaw boundary. By comparing the slope displacement before and after freeze–thaw, we can conclude that the sliding surface of the slope after freeze–thaw is mainly damaged by sliding in the freezing area. This is due to the freezing and thawing effect destroys the soil structure, resulting in the reduction of the shear strength of the bank slope soil, after freezing and thawing the bank slope soil is more prone to deformation, so when the temperature rises in the spring and the bank slope soil thawing, after experiencing the freezing and thawing erosion of the bank slope in the hydrodynamic scouring effect is more prone to damage.

##### Change in safety factor

**Figure 15:**
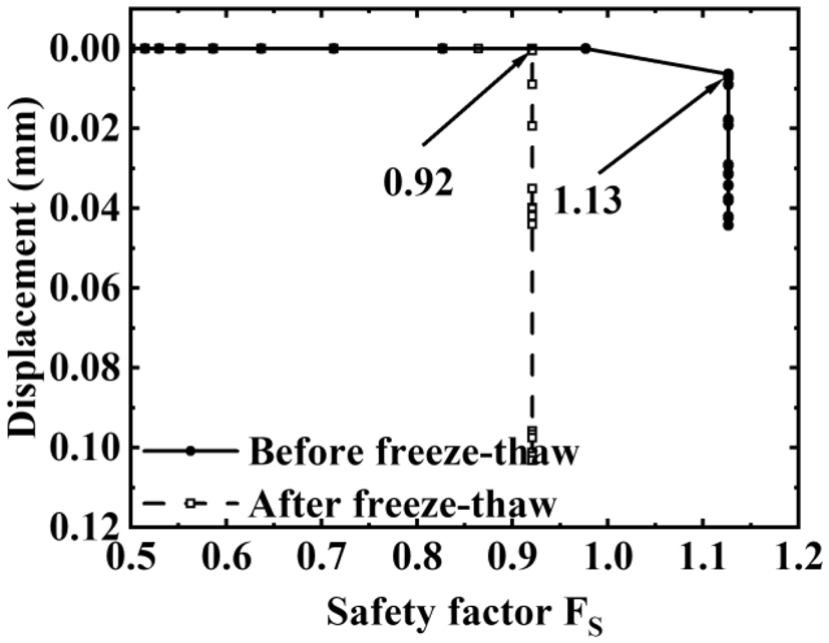
Safety factor of bank before and after freeze and thaw.

Figure 15 illustrates the curve of the coefficient of safety before and after freezing. It can be seen that the safety factor of 60° bank slope before freezing and thawing is 1.13, while it decreases to 0.92 after freezing and thawing. Generally^35^, when F_S_ > 1.3, the slope remains stable, when F_S_ < 1.0, the slope is damaged, and when 1.0 ≤ F_S_ ≤ 1.3, the slope may be damaged^25^, hence, the 60° slope is susceptible to destabilization damage after exposure to frost. Additionally, When the 60° bank slope is affected by freeze–thaw cycles, the safety factor of bank stability decreases by 18.58%, and the freeze–thaw action destroys the overall stability of the slope.

## Discussion

Freeze–thaw action destroys the soil structure and changes the soil properties, so the stability of riverbanks in seasonal permafrost zones has to consider freeze–thaw action. It can be found from this test that the strength parameter of the bank soils is significantly reduced after freeze–thaw action, and the more the number of freeze–thaw cycles, the greater the decrease of soil strength after freeze–thaw, which is the same as the findings of Tang et al.^12^ and Xu et al.^36^. This is because Soil consists of solid–liquid-gas three-phase, when the temperature drops below 0 ℃ in autumn, the moisture in the soil also freezes into ice, and the volume of moisture expands by about 9%, the soil particles are extruded, deformed and ruptured under the freezing and expansion effect of moisture, resulting in the emergence of new pore space and the continuous penetration of the original pore space, and the porosity of the soil body increases significantly under the action of freeze–thaw,^37^. After freezing and thawing, the large pores of the soil decrease and the small pores increase. The internal friction angle of soil mainly reflects the friction force and bite force between soil particles, and the cohesion reflects various physical and chemical forces between soil particles, including Coulomb force, van der Waals force and cementation force. The increase of the porosity of the soil will destroy the cementation between the soil and reduce the friction between the particles. Therefore, the freeze–thaw action will significantly reduce the cohesion and internal friction angle of the soil. In addition, the shear strength of the soil is calculated by the cohesion and internal friction angle of the soil, so the shear strength of the soil is also reduced by the freeze–thaw effect. However, Wang et al.^38^ through freeze–thaw triaxial tests on silt clay, found that after freeze–thaw, the cohesion of the soil body decreased, and the internal friction angle increased. The results differed significantly from those in this paper, mainly due to the differences in the grain size of the test samples. The study suggests that the grain size of soil particles is the determining factor for strength parameters^39^. The main component of silt clay is colloidal particles, whose grain size is significantly smaller than that of silt, which means that small particles may enter the large pores of the silt after freeze–thaw and play a filling role. This could increase the shear force between the soil particles, resulting in a larger internal friction angle of the silt clay soil body after freeze–thaw.

Furthermore, we can also find that the lower the freezing temperature, the greater the decrease in the strength of the soil, which is the same as the results of the study by Sun et al.^40^. This is because the freezing process, the lower the temperature, the more water frozen as ice in the soil, the more significant the effect of freezing and expansion of soil particles by water, the greater the porosity of the soil^41^^,^^42^, so the decline in soil cohesion, internal friction angle and shear strength will be greater. However, based on the study of Chang et al.^21^ on Tibetan sand, it appears that the impact of freezing temperature on the soil’s mechanical properties is relatively minor, without any definite pattern. This may be because coarse sand has a larger particle size, and its inherent porosity is already relatively high. Therefore, the influence of different freezing temperatures on soil porosity is relatively minor. The shear strength of the soil is the primary factor affecting slope stability. After freeze–thaw, the cohesion and internal friction angle of the slope’s sandy silt decrease, reducing the slope’s shear strength. This can lead to the destruction of slope stability, making it more prone to collapse during the spring thaw^29^.

## Conclusion

In this paper, the mechanical property changes of soil under different freezing temperatures and number of freeze–thaw cycles were investigated by freezing triaxial shear tests at different freezing temperatures. Based on the parameters of the triaxial freeze–thaw tests, the stability changes of the bank before and after freeze–thaw erosion were simulated and analyzed using the finite element method. Based on our findings, we came to the following conclusions:

1. The elastic modulus, cohesion, internal friction angle and shear strength of soil decrease after freeze–thaw action. . After 10 freezing cycles at − 5 ℃, − 10 ℃, − 15 ℃ and − 20 ℃, the modulus of elasticity of soil decreased by 40.84%, 47.53%, 35.96% and 68.70%, the cohesion decreased by 41.96%, 45.94%, 49.69% and 56.66%, the shear strength decreased by 41.92%, 46.25%, 50.54% and 57.32%, respectively.
2. The slope is damaged by circular sliding, and the slope begins to slide along the freeze–thaw boundary after freezing and thawing. The safety factor of slope decreases by 18.58% after freezing and thawing, and the freeze–thaw effect significantly reduces the stability of the slope, making it more susceptible to damage caused by instability after freezing and thawing.

Specifically, this study examines the effects of different freezing temperatures on the mechanical properties of slope soils and the stability of slopes in the Inner Mongolia section of the Yellow River. In the analysis of slope stability, only the freeze–thaw effect is considered. Future studies should examine the coupled effects of hydraulic erosion, water level change, waves, and other damage factors.

## Data Availability

The data used to support the findings of this study are included within the article, it can be provided by the author Yang Zhen.
